# Advanced Urothelial Carcinoma: Overcoming Treatment Resistance through Novel Treatment Approaches

**DOI:** 10.3389/fphar.2013.00003

**Published:** 2013-02-06

**Authors:** Richard M. Bambury, Jonathan E. Rosenberg

**Affiliations:** ^1^Memorial Sloan Kettering Cancer Center, Weill Cornell Medical CollegeNew York, NY, USA

**Keywords:** urothelial cancer, bladder cancer, oncogenes, chemotherapy, resistance mechanisms

## Abstract

The current standard of care for metastatic urothelial carcinoma is cisplatin-based chemotherapy but treatment is generally not curative. Mechanisms of resistance to conventional cytotoxic regimens include tumor cell drug efflux pumps, intracellular anti-oxidants, and enhanced anti-apoptotic signaling. Blockade of signaling pathways with small molecule tyrosine kinase inhibitors has produced dramatic responses in subsets of other cancers. Multiple potential signaling pathway targets are altered in Urothelial carcinoma (UC). Blockade of the PI3K/Akt/mTOR pathway may prove efficacious because 21% have activating PI3K mutations and another 30% have PTEN inactivation (which leads to activation of this pathway). The fibroblast growth factor receptor 3 protein may be overactive in 50–60% and agents which block this pathway are under development. Blockade of multiple other pathways including HER2 and aurora kinase also have potential efficacy. Anti-angiogenic and immunotherapy strategies are also under development in UC and are discussed in this review. Novel therapeutic approaches are needed in UC. We review the various strategies under investigation and discuss how best to evaluate and optimize their efficacy.

## Introduction

Urothelial carcinoma (UC) affects an estimated 76,000 people in the USA each year and causes 16,000 deaths (Siegel et al., 2012). The majority of cases are localized to the urinary tract but 25% present with or develop locally advanced or metastatic disease which is generally incurable (Bellmunt and Petrylak, 2012). The current standard of care for metastatic disease is combination chemotherapy with cisplatin and gemcitabine. This regimen was found to be equally effective but less toxic than the quadruplet methotrexate/vinblastine/doxorubicin/cisplatin (MVAC) regimen which had been the previous standard (von der Maase et al., 2000). Cisplatin/gemcitabine had a response rate of 49%, median progression free survival of 8 months, overall survival of 14 months, and 13% of patients survived ≥5 years. The median duration of response was 10 months but a minority of patients did achieve a durable complete remission (von der Maase et al., 2005).

## Mechanisms of Resistance to Cisplatin Chemotherapy

Cisplatin is the cornerstone of chemotherapy for metastatic UC. It acts by binding to purine DNA bases, forming inter- and intra-strand crosslinks, causing DNA damage, and thus activating the apoptotic pathway leading to cell death (Drayton and Catto, 2012). Multiple mechanisms of UC resistance to cisplatin have been identified and can be broadly classified into tumor cell drug efflux pumps, intracellular anti-oxidants, DNA repair pathway modulation, and enhanced anti-apoptotic signaling (Drayton and Catto, 2012).

The main mechanism of cisplatin efflux from the cell is by the ATP7A and ATP7B proteins (Komatsu et al., 2000; Samimi et al., 2004). Increased expression of these proteins is associated with cisplatin resistance in some tumor types including ovarian cancer (Samimi et al., 2004). However, in UC it appears that increased cisplatin efflux is not a major mechanism of resistance and accordingly a recent study in UC cell lines showed no correlation between levels of intracellular cisplatin and sensitivity to the drug (Yu and Wang, 2012).

Intracellular binding and sequestration of cisplatin by thiol proteins like metallothioneins and glutathione can also neutralize its activity (Drayton and Catto, 2012). Increased expression of both metallothioneins and glutathione has been correlated with UC resistance to cisplatin in cell line studies (Siegsmund et al., 1999; Byun et al., 2005). At a clinical level, absence of UC metallothionein expression by immunohistochemistry and low levels of glutathione as measured by liquid chromatography are correlated with response to neoadjuvant cisplatin-based chemotherapy (Bahnson et al., 1994; Yang et al., 1997).

ERCC1 is a protein involved in repairing DNA which is damaged by cisplatin. Increased expression in cancer cells is correlated with shorter survival after treatment with cisplatin-based chemotherapy (Bellmunt et al., 2007). Defects in the ability of cancer cells to recognize DNA damage and undergo apoptosis also play a role in cisplatin resistance. Bcl-2 is an anti-apoptotic protein which is upregulated in UC and in cell line studies knock-down of Bcl-2 expression rendered previously resistant cells sensitive to cisplatin (Hong et al., 2002). p53 is the most commonly mutated gene in human cancers and cell line studies have found that these mutations may increase cisplatin resistance in UC (Drayton and Catto, 2012). However, attempts to use this finding for treatment stratification in the clinic have so far proved fruitless (Stadler et al., 2011).

Mechanisms of UC resistance to gemcitabine have been less well studied but putative factors include upregulation of heme-oxygenase 1 and the anti-apoptotic protein clusterin (Muramaki et al., 2009; Miyake et al., 2010). Gemcitabine resistance has been more extensively investigated in other cancers and researchers have found resistance associated with upregulation of heat shock protein 27 (further discussed below) and multidrug membrane transport proteins (ABCG2 and ABCA9) in pancreatic cancer and upregulation of bcl-2 in breast and gastric cancer (Kuramitsu et al., 2012; Van den Broeck et al., 2012; Wong et al., 2012).

## Efforts to Improve the Efficacy of Cytotoxic Chemotherapy

In an effort to improve outcomes with cisplatin/gemcitabine a phase 3 trial assessed the addition of paclitaxel to this regimen (Bellmunt et al., 2012). While response rate was improved from 44 to 56% with the addition of paclitaxel, there was no significant improvement in overall survival. An analysis of patients in this trial with tumors of bladder origin (rather than upper tract UC) did reveal a statistically significant survival improvement although this was a *post hoc* subset analysis (Bellmunt et al., 2012).

Another strategy to improve outcomes with conventional chemotherapy is dose intensification using growth factor support. Human cancer cells grow by Gompertzian kinetics whereby growth rates decrease with increasing tumor size (Norton, 1988). Dose-dense scheduling is designed to capitalize on this phenomenon by delivering successive cycles of chemotherapy at shorter intervals when the residual tumor burden is smaller, faster growing, and hence more chemosensitive (Morris et al., 2010). It also allows higher cumulative doses of chemotherapy to be administered in a given time-frame. The approach has been successful in the adjuvant treatment of breast cancer, although did not improve outcomes in diffuse large B cell lymphoma (Citron et al., 2003; Cunningham et al., 2011). In metastatic UC, a phase 3 clinical trial evaluated classic MVAC given every 28 days against a dose-dense MVAC regimen administered every 14 days with granulocyte colony stimulating factor support. There was no significant median overall survival benefit with the dose-dense approach in this study. However response rates were improved and a larger number of patients did appear to gain long term disease remission [5 year progression free survival (16.5 vs. 8%) and overall survival (22 vs. 14%) favoring intensified chemotherapy; Sternberg et al., 2006]. Dose intensified gemcitabine/cisplatin appears to be a promising alternative with a lower toxicity profile based on preliminary reports of an aborted phase III trial (Bamias et al., 2012). This regimen is being further tested in the neoadjuvant setting (NCT01589094 and NCT01611662).

Heat shock proteins are a class of proteins which are upregulated during cellular stress. Among their numerous functions include acting as “molecular chaperones” to stabilize signaling molecules which may include oncogenic proteins (Richardson et al., 2011). In UC, HSP 70-2 is over-expressed and knock-down of its expression in xenograft studies suppressed tumor growth (Garg et al., 2010). Recent mouse model work has shown the potential of a HSP 90 inhibitor to overcome cisplatin resistance (Tatokoro et al., 2012). HSP 27 is also implicated in UC chemoresistance and an anti-sense oligonucleotide targeting its expression is currently under active investigation in a randomized phase 2 trial combining it with chemotherapy (NCT01454089; Kamada et al., 2007; Hadaschik et al., 2008).

Another route to improving the effectiveness of cytotoxic chemotherapy in UC is to prospectively identify those patients most likely to respond. This would maximize the benefits from chemotherapy and spare many patients unnecessary toxicity. To this end, identification and validation of potential predictive tumor biomarkers such as ERCC1, Ribonucleotide reductase subunit M1 (RRM1), BRCA1, and miR-34a is warranted (Chang et al., 2012). In addition, germline polymorphisms may provide information about the likelihood of response to a given treatment. In UC, Gallagher et al. (2011a) identified four single nucleotide polymorphisms (SNPs) which predicted a likelihood of response to chemotherapy varying from 19 to 84%. A composite germline and somatic genetic signature could prove to be more predictive than either one alone (Bambury and Gallagher, 2012).

## Novel Approaches to the Treatment of Metastatic Urothelial Cancer may Overcome Therapeutic Resistance

### Signaling pathway blockade

**Figure 1:**
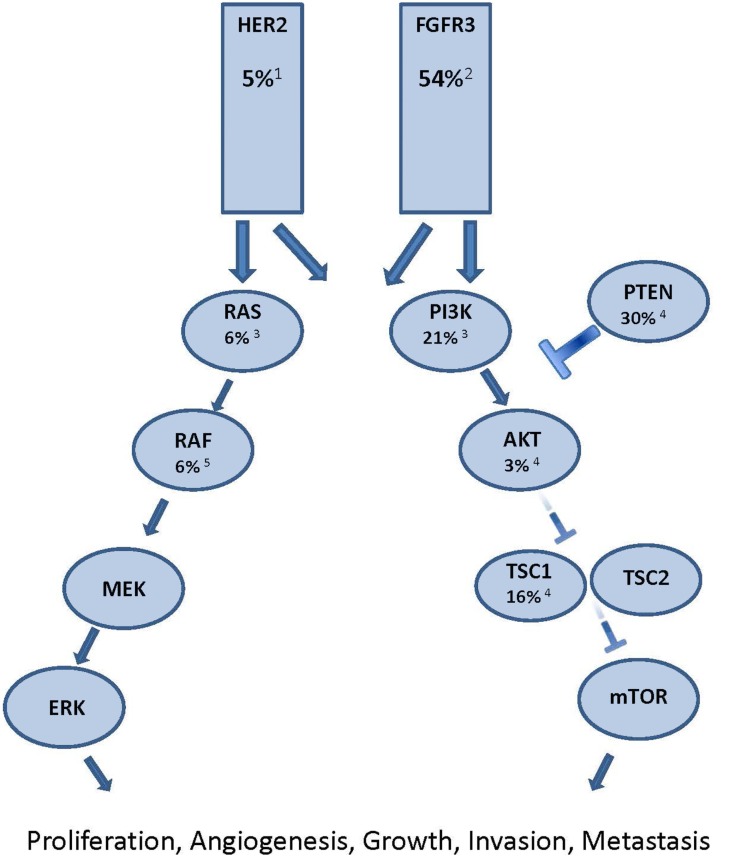
**Major oncogenic signaling pathway alterations in urothelial carcinoma**. Each potential target is identified with the proportion of muscle invasive UC known to have oncogenic alterations in the signaling molecule. Superscript denotes reference article. ^1^Lae et al., 2010; ^2^Tomlinson et al., 2007a; ^3^Kompier et al., 2010; ^4^Ching and Hansel, 2010; ^5^Boulalas et al., 2009

Of the thousands of genetic alterations in a given cancer cell there are likely to be only a few “driver” mutations which have a significant pro-neoplastic effect (Torti et al., 2012). The growth and survival of some cancers is dependent on the continued over-activity of certain signaling pathways due to these driver mutations, which is known as “oncogene addiction” (Weinstein, 2002). Small molecule tyrosine kinase inhibitors (TKIs) block signaling through the relevant pathway and induce cell-cycle arrest, differentiation, or apoptosis (Torti and Trusolino, 2011). Clinical examples of the implementation of this principle for patient benefit include using imatinib to block bcr-abl signaling in chronic myeloid leukemia and vemurafenib to block mutated BRAF signaling in melanoma (O’Brien et al., 2003; Chapman et al., 2011). While the clinical benefit of targeting oncogenic activating mutations has not been proven in UC, there are multiple potential molecular targets with tantalizing hints of efficacy beginning to appear (Figure 1; Iyer et al., 2012).

The PI3K/Akt/mTOR pathway is one potential therapeutic target. Approximately 21% of muscle invasive UC have activating PI3K mutations, while another 30% demonstrate evidence of PTEN inactivation (Ching and Hansel, 2010; Kompier et al., 2010). In addition, 16% of patients have inactivating mutations in TSC1, an inhibitor of mTOR activation. Indeed, a recently published report in *Science* describes a durable complete response to everolimus in a patient with chemotherapy refractory metastatic UC in which an inactivating mutation in the TSC1 gene was identified. In that study, four of five patients with TSC1 mutation experienced objective tumor shrinkage, compared with one of nine patients without TSC1 mutation. These findings raise the possibility that mTOR inhibition may be an effective therapeutic strategy for the subset of metastatic UC patients with genetic activation of the PI3K/Akt/mTOR pathway. However, in unselected patients, everolimus showed disappointing results, with a 5% response rate (Seront et al., 2012). Retrospective analysis of this trial revealed that presence of PI3K mutations did not necessarily correlate with response, highlighting the fact that therapeutic manipulation of molecular pathways may be more complex than simply targeting tumors with evidence of mutational pathway activation (Seront et al., 2012). In another study enrolling patients of all tumor types in early phase clinical trials of regimens including PI3K/AKT/mTOR inhibitors, the authors found a 17% response rate in patients with known PI3K pathway alteration (as evidenced by PIK3CA mutations, PTEN aberrations, or both) compared with 6% in those without (Janku, 2012). Furthermore, the presence of a synchronous activating k-ras mutation decreased likelihood of response to 4% and treatment with monotherapy had an inferior response rate (2.5%) compared with treatment using dual pathway blockade (23%). Future trials enriching for potential responders by accruing patients based on mutational profiles may improve the therapeutic index.

The *ERBB2* gene encoding the HER2 protein is amplified in approximately 5% of UC (Lae et al., 2010). A single arm phase II study of patients with Her2 positive UC (defined as 2+ or 3+ by immunohistochemistry, positive FISH, or elevated serum Her2/*neu* extracelluar domain) showed an encouraging 70% response rate with the addition of trastuzumab to paclitaxel, gemcitabine, and carboplatin (Hussain et al., 2007). Results are awaited from a European randomized phase 2 study comparing standard chemotherapy with or without trastuzumab in Her2 positive metastatic UC (Beuzeboc et al., 2007). In addition, the ongoing phase II/III LaMB UK study is randomizing patients with HER1 and/or HER2 overexpressing locally advanced or metastatic bladder UC to maintenance lapatinib vs. placebo in patients with stable or responding disease after first-line chemotherapy (NCT00949455).

The fibroblast growth factor receptor 3 (FGFR3) is another transmembrane receptor tyrosine kinase which harbors activating mutations in UC (Iyer and Milowsky, 2012). It has activating point mutations or amplifications in 50–60% of muscle invasive bladder UC (Figure 1). Activating point mutations are more commonly found in superficial bladder UC than the muscle invasive phenotype (van Rhijn et al., 2010). Cell line and xenograft studies have shown the anti-cancer effects of inhibiting mutant FGFR3 (Tomlinson et al., 2007b). Multiple agents which block this pathway are under active development and the most clinically advanced of these is the Novartis agent TKI12458. Results are awaited from a phase II trial (NCT00790426) of this agent as second and third-line therapy for patients with advanced UC. Patients were stratified by the presence or absence of FGFR3 activating point mutations to determine whether there is predictive and therapeutic utility in targeting this pathway. In addition to activating point mutations and amplifications, a recent report describes oncogenic FGFR3 genetic translocations and re-arrangements as an alternative mechanism of pathway activation (Williams et al., 2013). Another study observed FGFR3 overexpression by immunohistochemistry in 42% of FGFR3 wild-type tumors (Tomlinson et al., 2007a). Whether some or all UC tumors with FGFR3 activating point mutations, gene amplifications, gene re-arrangements, or immunohistochemical overexpression will respond to pathway inhibition remains to be determined but investigation of such an approach is certainly warranted.

Activating mutations of the *BRAF* gene are known to be present in 7–8% of all cancers and are present across a wide range of tumor types (Flaherty et al., 2010). Vemurafenib, an orally administered agent which specifically blocks V600E mutated BRAF, is highly active in patients with V600E BRAF mutant metastatic melanoma (Chapman et al., 2011). It has also shown activity in reported cases of lung cancer and leukemia with this mutation (Dietrich et al., 2012; Gautschi et al., 2012). Data on the frequency of BRAF mutations in UC are sparse but one small study detected mutations in 2 of 30 patients studied, of which one was a V600E mutation (Boulalas et al., 2009). Vemurafenib may prove to be an active agent in selected UC cases bearing BRAF mutations.

### Anti-angiogenic therapy

As one of the seven hallmarks of cancer, angiogenesis is known to play a critical role in the development and proliferation of all malignancies (Hanahan and Weinberg, 2011). UC tumors with increased angiogenesis as measured by higher microvessel density or higher serum levels of vascular endothelial growth factor (VEGF) have been shown to have worse prognosis (Bochner et al., 1995; Bernardini et al., 2001).

Bevacizumab is a monoclonal antibody directed against circulating VEGF. It has shown clinical benefit in colorectal, lung, and other cancers. A phase II trial of cisplatin/gemcitabine and bevacizumab as first-line therapy for metastatic UC revealed an encouraging overall response rate of 72% and an overall survival of 20.4 months (Hahn et al., 2011). To further investigate this promising finding, a phase III study is currently accruing which randomizes patients to standard therapy with cisplatin/gemcitabine ± bevacizumab (NCT00942331).

Sunitinib is a multi-targeted TKI with activity against ?VEGFR, PDGFR, Kit, FLT3, and RET. It was studied in the first-line setting combined with gemcitabine/cisplatin but the combination was found to be associated with severe hematologic toxicity (Galsky et al., 2010). Two phase II trials have evaluated its use as single agent therapy (one as first-line treatment in cisplatin ineligible patients and the other as second-line treatment) with response rates of 5–8% reported (Bellmunt et al., 2011; Gallagher et al., 2011b). Pazopanib is a TKI against ?VEGFR, PDGFR, and Kit. It was studied as a single agent in a phase II trial in chemo-refractory advanced UC and demonstrated a 17% response rate, although the results of this study are difficult to interpret due to the non-standard evaluation schedule used (Necchi et al., 2012).

### Immunotherapy

Adjuvant intravesical immunotherapy with Bacillus Calmette–Guerin (BCG) for non-muscle invasive bladder cancer is a standard therapy, and demonstrates the importance of immune stimulation in the treatment of UC (Hussain et al., 2009). Based on, investigation of immune checkpoint inhibitors in UC is warranted. For example, CTLA-4 blockade with ipilimumab has shown efficacy in metastatic melanoma (Hodi et al., 2010). An exploratory study of its use in the neoadjuvant setting for 12 patients with bladder UC showed it to be well tolerated. There was increased frequency of CD4 + ICOS high T cells in tumor tissues and in the systemic circulation after ipilimumab suggesting that an anti-tumor immune response was induced, although the clinical relevance of this finding is as yet unknown (Carthon et al., 2010). Sipuleucel-T is an autologous active cellular immunotherapy which improves survival in metastatic castrate refractory prostate cancer (Kantoff et al., 2010). Using the same platform to activate peripheral-blood mononuclear cells against Her2 expressing UC cells, an ongoing randomized phase II study is investigating the efficacy of DN24-02 in Her2 positive UC in the adjuvant setting (NCT01353222). There are also a number of anti-tumor vaccines under investigation in UC targeting antigens such as human chorionic gonadotropin-beta (β-hCG) and NY-ESO-1 (NCT00948961; Sharma et al., 2008; Morse et al., 2011).

## Conclusion

The development of precise anti-cancer agents which target known molecular aberrations presents a considerable challenge for clinical trial design. The accepted standard for demonstration of efficacy of new cancer therapies has been the phase III randomized controlled trial design. However enrolling patients to such trials in the new era of precision medicine will pose logistic and ethical challenges. The subdivision of patients into smaller and smaller groups based on detailed molecular analysis is re-defining cancer as “a thousand rare diseases” (Kerr, 2012). Accruing large numbers of patients to trials evaluating medicines that are targeted at small subsets will become increasingly difficult. In addition, a randomized trial design may deny or delay patients’ access to highly active medicines, which could be ethically questionable. One way to meet the challenge of accruing sufficient number of patients to trials of these new agents would be to allow enrollment of all patients who have the molecular profile expected to benefit from treatment regardless of cancer site of origin. Agents in these single arm trials of enriched patient populations which demonstrate high-levels of therapeutic activity should be considered for regulatory approval, with the proviso that careful post-marketing safety follow-up and ongoing trials are undertaken in large cohorts to validate the results.

To maximize the efficacy of new treatments, a combinatorial rather than single agent approach may be required, similar to combination cytotoxic chemotherapy for UC. Dual therapy with TKIs blocking different points in the same signaling pathway has improved efficacy over single agent therapy in metastatic melanoma (Flaherty et al., 2012). Similarly, synchronous blockade of parallel growth signaling pathways has improved efficacy in breast cancer (Baselga et al., 2012). Combinations of different therapeutic modalities such as chemotherapy with anti-angiogenic agents is also under investigation as discussed above.

Despite major strides in other malignancies, the treatment of advanced UC has made no major progress over the past 10 years. The ultimate goal of treatment for patients with metastatic cancer is to induce long term disease remission or stability. UC may be driven by alterations of different signaling molecules including HER2, FGFR3, and members of the PI3K pathway. Indeed, it may itself prove to be “a thousand rare diseases” with individual treatments dictated by the molecular profile of a given patients tumor. It is unclear which of the treatment strategies currently under investigation will be most beneficial for UC patients but the ongoing phase III study of bevacizumab added to conventional chemotherapy places this compound closest to the regulatory finishing line should the trial prove positive. Targeting specific signaling pathways along with pursuing the other avenues of research discussed above will hopefully lead us to new effective therapies for this lethal disease.

## Conflict of Interest Statement

Dr Jonathan E Rosenberg is a consultant for Oncogenex, Boehringer Ingelheim and Dendreon. Dr Richard M. Bambury declares that the research was conducted in the absence of any commercial or financial relationships that could be construed as a potential conflict of interest.
